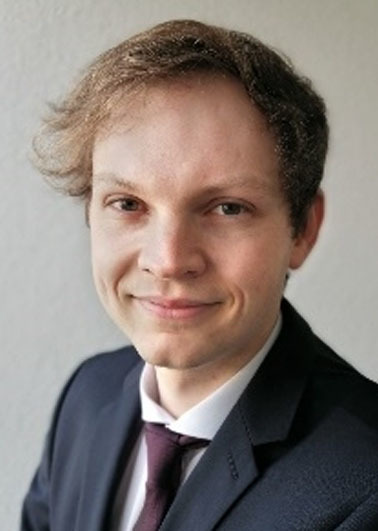# Using Light to Modify the Selectivity of Transition Metal Catalysed Transformations

**DOI:** 10.1002/anie.202105043

**Published:** 2021-06-17

**Authors:** Danijela Lunic, Enrico Bergamaschi, Christopher J. Teskey

**Affiliations:** ^1^ Institute of Organic Chemistry RWTH Aachen Landoltweg 1 52074 Aachen Germany

**Keywords:** divergence, photochemistry, photoswitching, selectivity, transition metal catalysis

## Abstract

Light has a remarkable and often unique ability to promote chemical reactions. In combination with transition metal catalysis, it offers exciting opportunities to modify catalyst function in a non‐invasive manner, most frequently being reported to switch on or accelerate reactions that do not occur in the dark. However, the ability to completely change reactivity or selectivity between two different reaction outcomes is considerably less common. In this Minireview we bring together examples of this concept and highlight their mechanistically distinct approaches. Our overview demonstrates how these non‐natural, photo‐switchable systems provide key fundamental mechanistic insights, enhancing our understanding and stimulating development of new catalytic activity, and how this might lead to tangible applications, impacting fields such as polymer chemistry.

## Graphical abstract

Light can be used to switch on or accelerate the catalytic activity of transition metals. However, using light to modify the selectivity of a transformation is considerably less common. This Minireview highlights early examples of controlling catalyst activity in this way and looks at the distinct approaches that have been taken to date.

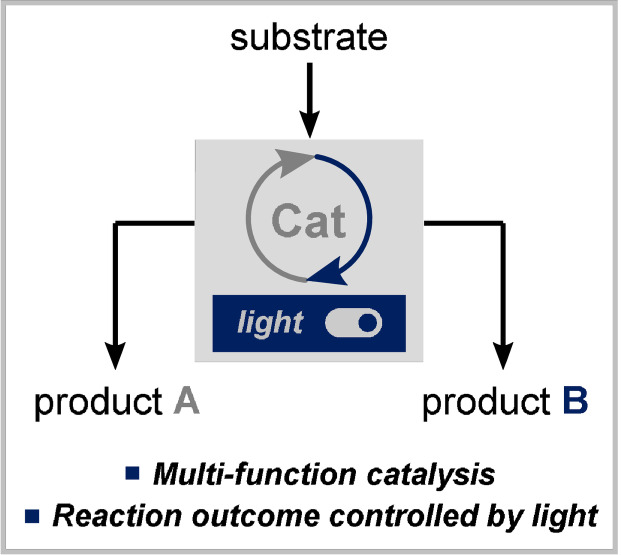

## 1 Introduction

Transition metal catalysis has revolutionised synthetic chemistry, leading to the development of entirely new reactivity, more‐efficient transformations and enabling access to fresh chemical space. At the same time, photochemistry has seen a resurgence of interest in the last decades, allowing access to radical reaction pathways under mild and controlled conditions. Unsurprisingly, the combination of these two fields is therefore currently an area of intense interest and a number of recent reviews have dealt with various aspects of this area, from dual catalysis[^1^, ^2^] to direct excitation of transition metal catalysts.[^3^, ^4^]

The major focus when developing catalytic systems has been to tune their properties to maximise conversions, enhance functional group tolerance and improve selectivities. However, once a new system has been developed, these characteristics are fixed, such that a reaction will have a preprogrammed outcome. Only then, by physically changing the reaction conditions (for instance the reagent, the catalyst, the ligand) is it possible to modify the enantio‐, diastereo‐, chemo‐ or regioselectivity of a reaction. Nevertheless, divergent strategies such as these are valuable because they allow chemists to obtain different products from one common precursor.^[5]^ Furthermore, the ability to selectively obtain different reaction outcomes on the same molecule can result in shorter and more efficient reaction sequences, and newly selective methods can give products that were previously inaccessible.

In contrast to these single‐function catalytic platforms, an area of research that has received significantly less attention is the development of multifunction transition metal catalysts. Here, the activity of the system can be switched by an external stimulus, allowing several different reaction outcomes to be obtained. Light is a particularly attractive stimulus to use, as it is non‐invasive and easily switchable, thus offering excellent spatio‐temporal control. However, most commonly, light is used only to switch on or accelerate a reaction.^[6]^ In this Minireview, we will focus on the examples in the literature where two different reaction outcomes can be obtained with the same transition metal catalyst, using light irradiation to dictate which product is formed, either by switching a light source on, modifying its wavelength or changing the intensity. Although the current number of examples is somewhat limited, already a broad range of strategies has been reported, offering exciting opportunities to modify catalyst function in a non‐invasive manner, and making the area ripe for further exploration.

**Figure 1:**
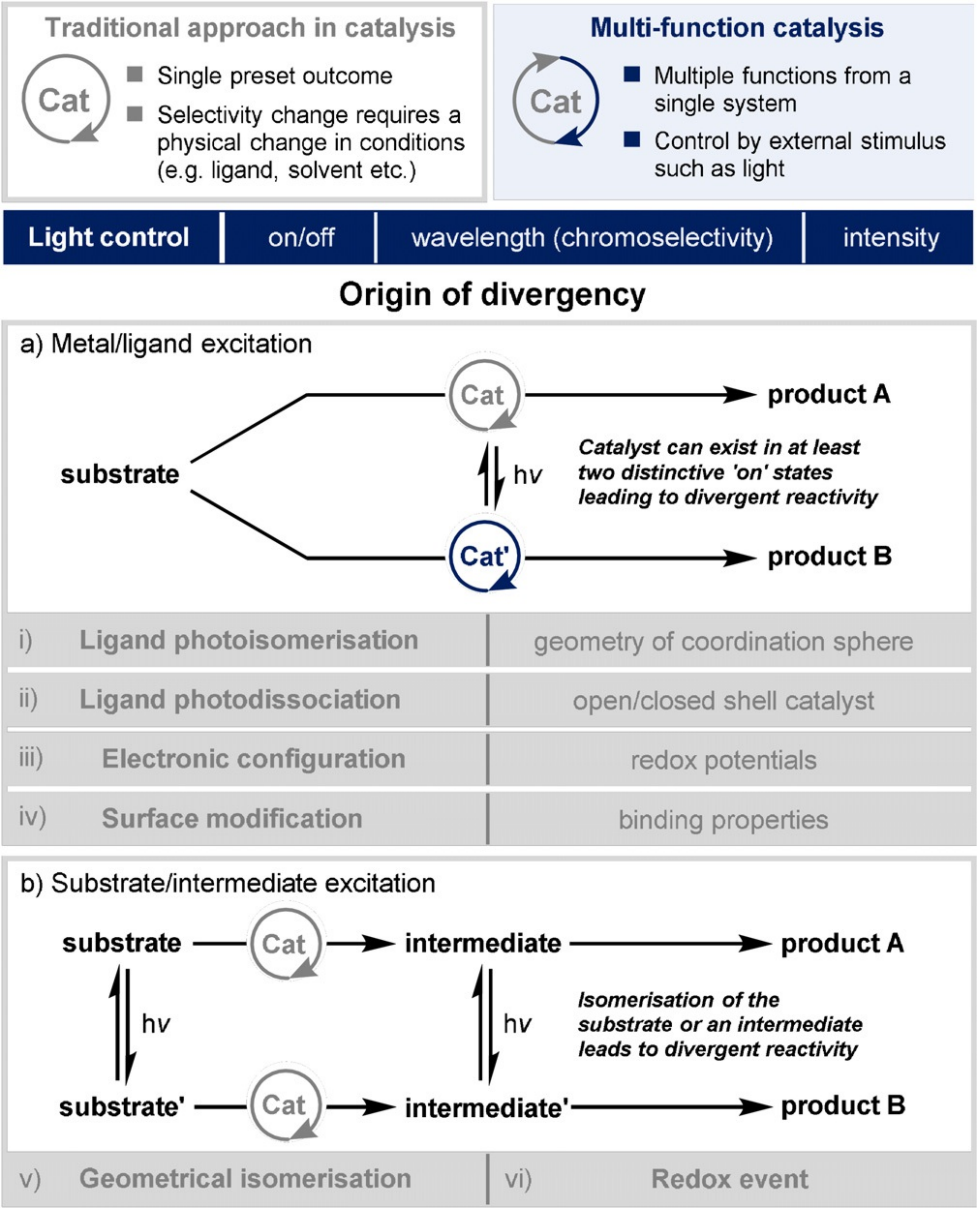
Summary of strategies for controlling the selectivity of transition metal catalysed reactions with light.

Broadly, current strategies can be split into two categories: light‐induced modification of either the catalyst or of the substrate (Figure 1 a,b). Where light is used to change the identity of the reactive catalytic species, this can involve photoisomerisation of the ligand structure which affects either the geometry (and thus the shape of the catalyst binding site) or the electronic properties at the metal centre. Alternatively, excitation of the transition metal complex can lead to ligand photodissociation, affecting the ability of the substrate to bind to the metal centre, which can result in divergent mechanistic pathways. Excitation of the catalyst may also result in alteration of the electronic configuration and its redox properties and, finally, particularly in heterogeneous catalysis, light irradiation can result in the alteration of the physical properties at the catalyst surface.

Alternatively, light can be used either directly or via a second catalyst to modify the starting material or a reactive intermediate, inducing either isomerisation or a redox event, such that the molecule undergoing the transformation by the transition metal is different, depending on the presence or absence of light irradiation.

The examples highlighted in this Minireview range from the exquisitely designed to those which reveal unexpected divergences of mechanism. Notably, it may not always be predictable which parts of the reaction that light will exert control over, or indeed whether the effect of light can necessarily be limited to only one aspect. Given the range of possibilities, it is entirely possible that future research may involve multiple divergency points in the mechanisms between light and dark. We therefore expect the number of light‐switchable transition metal catalysed processes to increase significantly in the years to come, opening up new avenues for on‐demand selective synthesis.

Excitingly, due to the widespread availability of LED light sources, more and more synthetic research groups may find themselves working in this area. Unexpected photoreactivity which contrasts with thermal reactivity of catalysts will almost certainly lead to the discovery of new modes of catalytic activity, expanding even further the reach of transition metal catalysis and allowing previously unattainable selectivities in chemical synthesis. Excellent mechanistic understanding, through spectroscopic and computational methods, will be essential to accompany developments in this area so that any discoveries can be fully capitalised on.

## 2 Catalyst Modification

### 2.1 Light‐Responsive Ligands

There has been significant interest in developing new molecular photoswitches that can be reversibly toggled between two thermally stable isomers.^[7]^ These molecular motifs possess at least two distinct structural geometries and have found potential applications in molecular devices. However, applications of these switches for regulating chemical reactivity of catalysts had been limited until recently.^[8]^ Several examples have now been reported which rely on carefully crafted systems, with two distinctive shapes, giving rise to active catalytic species with different steric or electronic properties at the reactive transition metal centre.

**Scheme 1:**
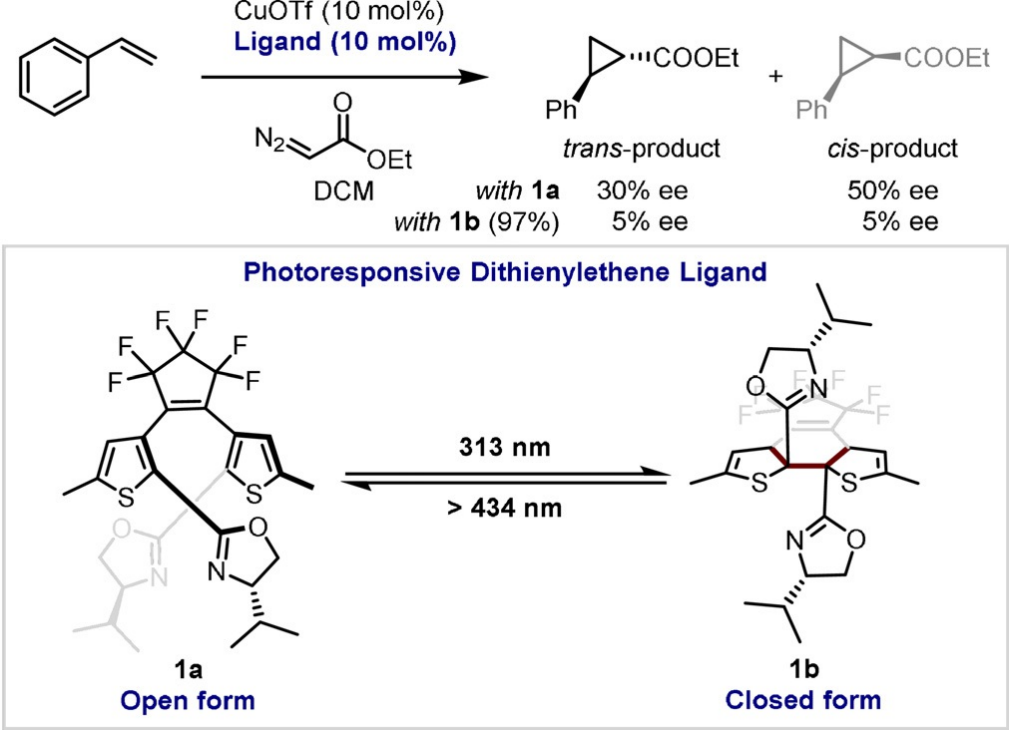
Photoresponsive ligands that control enantioselectivity.

One of the first examples to demonstrate a degree of photocontrolled selectivity in a transition metal catalysed transformation was reported by Branda and co‐workers in 2005.^[9]^ Here, the authors reported the careful design of a bis(oxazoline) ligand which incorporates a 1,2‐dithienylethene (DTE) moiety into the backbone. The significant change in the metal‐binding pocket of the ligand between ring‐opened (**1 a**) and ring‐closed (**1 b**) forms was demonstrated in the context of a copper‐catalysed cyclopropanation reaction (Scheme 1). The ring‐opened form of the ligand yielded the products with moderate enantiomeric excess (*ee*); however, this was completely eroded upon UV irradiation, which ring‐closes the DTE moiety of the bis(oxazoline) ligand.

Whilst this case demonstrated the loss of selectivity of a transition metal catalyst, the first example of a complete switch of selectivity, such that a single enantiomer of a ligand could be used to obtain both enantiomers of a product on demand, was reported by the Feringa group in 2015.^[10]^ They designed the chiral phosphine ligand **2 a**–**d**, inspired by other *C*_2_‐symmetric ligands, which could exist in two (pseudo‐)enantiomeric forms owing to the incorporation of a crowded alkene molecular motor core. Notably, this four‐stage switch also allows access to the racemic structures.

**Scheme 2:**
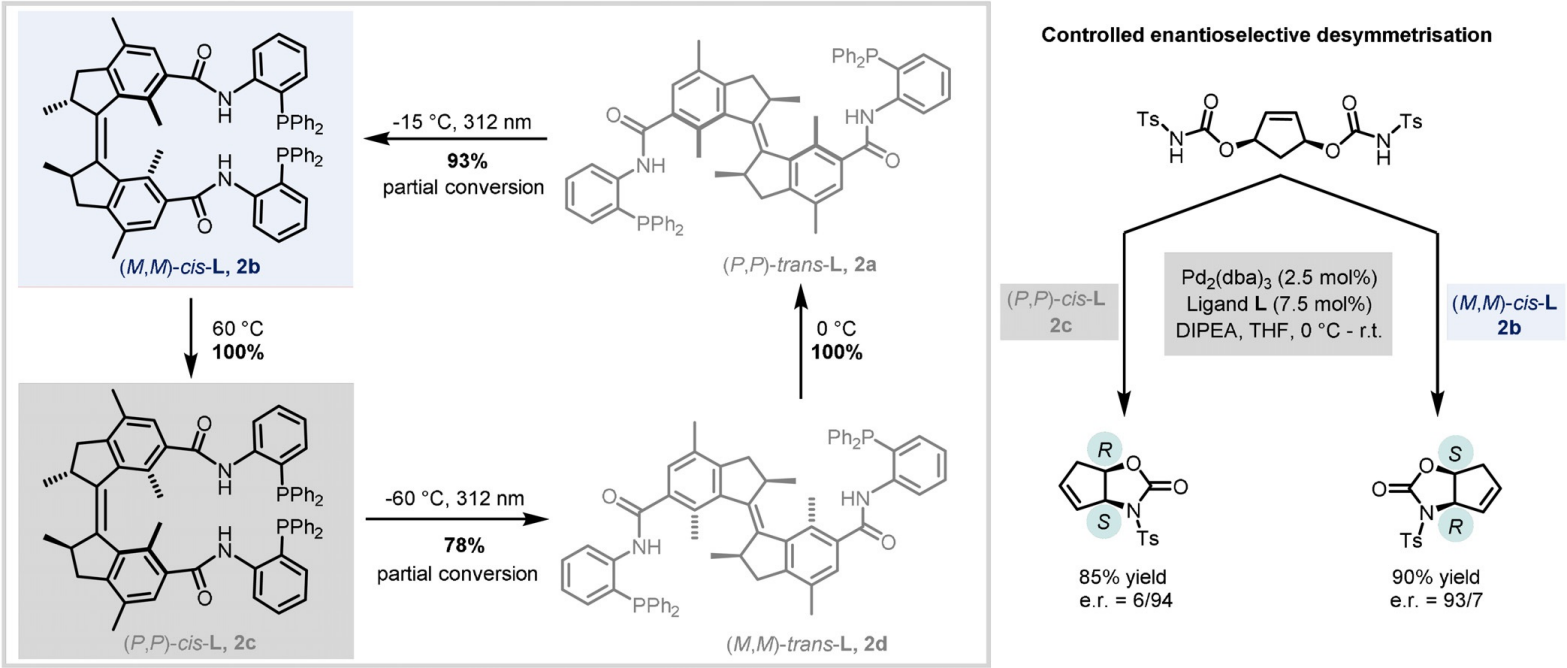
Controlling enantioselectivity in palladium‐catalysed desymmetrisation reactions using ligands that incorporate molecular motors.

The devised catalyst system, using the chiral switchable bisphosphine ligands, was applied for the enantioselective control of Pd‐catalysed desymmetrisation reactions of biscarbamates. Dual stereocontrol is achieved by the use of a unidirectional light‐driven molecular motor with an amide linker which undergoes a 360° four‐stage rotary cycle, involving photo‐ and thermal helix isomerisation steps, and leading to the change of helicity and geometry of the ligand (Scheme 2). Excellent enantioselectivity is attained by the stepwise control of the rotation cycle, which leads to the formation of three stable ligand isomers: *P*,*P*‐trans **2 a** catalysing the formation of racemic mixture, and pseudo‐enantiomers *M*,*M*‐cis **2 b** and *P*,*P*‐cis **2 c**, both of which lead to the formation of a single enantiomer of oxazolidinone (Scheme 2). However, thermally induced helix inversion between the pseudo‐enantiomeric isomers is irreversible, and in order to recover the initial **2 b**, three consecutive isomerisations (light–heat–light) are necessary, instead of one isomerisation starting from the *P*,*P*‐cis form.

**Scheme 3:**
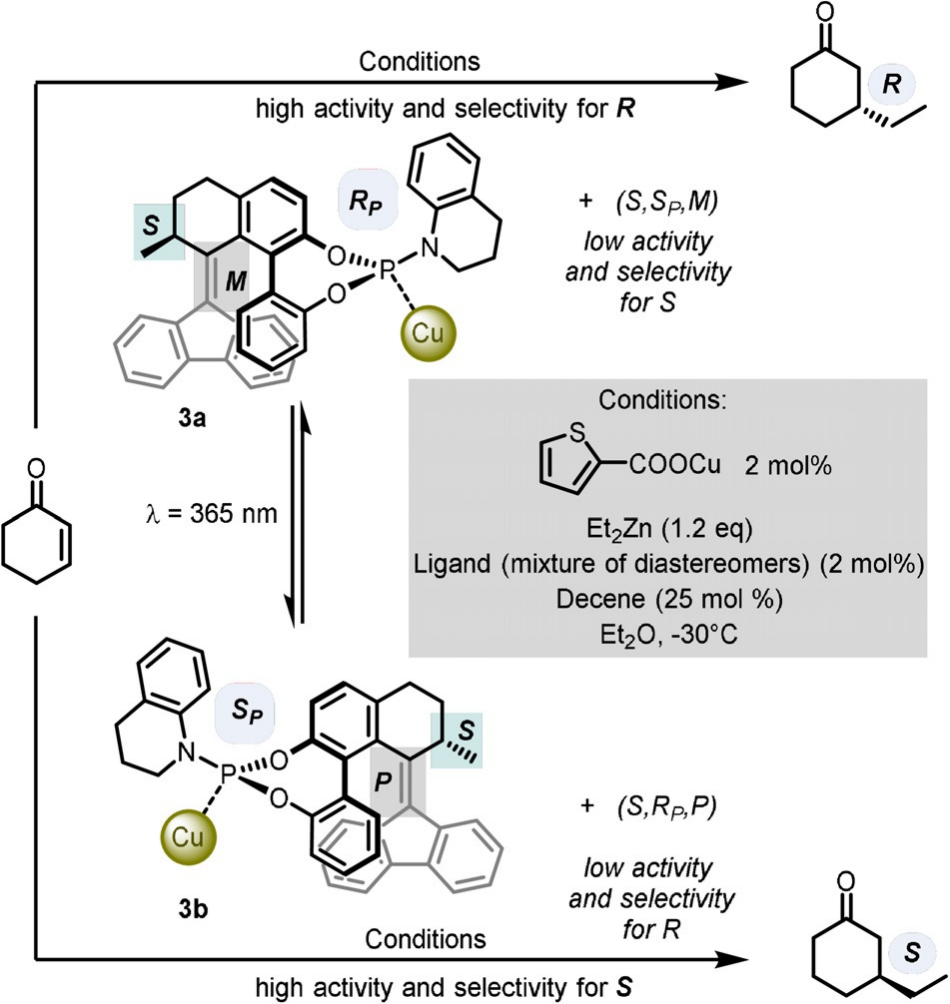
Enantioselective control of conjugate addition using precisely designed phosphoramidite ligands.

More recently, the Feringa group reported the development of a system with phosphoramidite molecular switches based on second‐generation overcrowded alkene molecular motors.^[11]^ The switch core contains a six‐membered ring in the upper half and a five‐membered ring in the lower half (Scheme 3, **3 a** and **3 b**). Compared to the previous motor, there are only two isomerisation stages, since the biphenyl motif suppresses the continuous unidirectional rotation about the central alkene. Furthermore, the photogenerated metastable states possess long half‐lives (up to 1.3 years at 20 °C), meaning both pseudo‐enantiomers of the switch can be used without continuous irradiation over longer time periods.

The design of the ligand is based on combining the photoswitchable biphenol motif with an amine at the phosphorus centre to make up the phosphoramidite. The crowded alkene can be atropisomerised without disrupting the bridged biaryl unit or affecting its flexibility, resulting in a significant change of geometry and steric environment around the phosphorus centre. In the phosphoramidite synthesis, a mixture of diastereomers at the phosphorus centre are also obtained, depending on the identity of the amine. This turns out to be important for the effective switching of enantioselectivity, because the barrier for pyramidal inversion at the phosphine is high enough to prevent it at room temperature.

In total, the ligands have five stereochemical elements: the fixed phosphorus configuration (Scheme 3, pale blue box, *R_P_* or *S_P_*); the fixed carbon stereogenic centre (Scheme 3, green box, *S*); and then three interconnected elements—the helicity of the overcrowded alkene, the dynamic helical geometry and the axial chirality of the biaryl unit—which are given a combined stereodescriptor based on the helicity of the alkene chromophore (Scheme 3, grey box, *M* or *P*).

These newly developed ligand systems were studied in the context of copper(I)‐catalysed asymmetric conjugate additions of diethylzinc to 2‐cyclohexen‐1‐one. Reversible switching of chirality is achieved by light: irradiation at 365 nm triggers isomerisation of the ligand and causes a change of catalytic activity and enantioselectivity. Unlike previous examples of ligands containing alkene photoswitches, where photoisomerisation was possible only with the free ligand (due to quenching of the alkene excited state by energy transfer to the metal), this system allows dynamic switching even with the ligand bound to the copper centre. Interestingly, the overall catalytic performance of the ligand structure is dictated by the matched and mismatched relationship between the fixed chirality at the phosphorus and the dynamic helicity of the photoswitch. Therefore, the most effective system for significant changes in enantioselectivity were those that contained two competing diastereomeric ligands in the two reversible states of the system. For the ligand system shown in Scheme 3, prior to irradiation, the mixture consists of the poorly active major diastereoisomer (*S*,*S_P_*,*M*) with stereoselectivity for the (*S*)‐product and the highly active minor diastereoisomer **3 a** (*S*,*R_P_*,*M*) with stereoselectivity for the (*R*)‐product. After photoswitching, the behaviour is inverted: the weakly active major isomer becomes more active to give **3 b** (*S*,*S_P_*,*P*), and the very active minor isomer is converted into its less active counterpart (*S*,*R_P_*,*P*), therefore overall favouring formation of the (*S*)‐product.

**Scheme 4:**
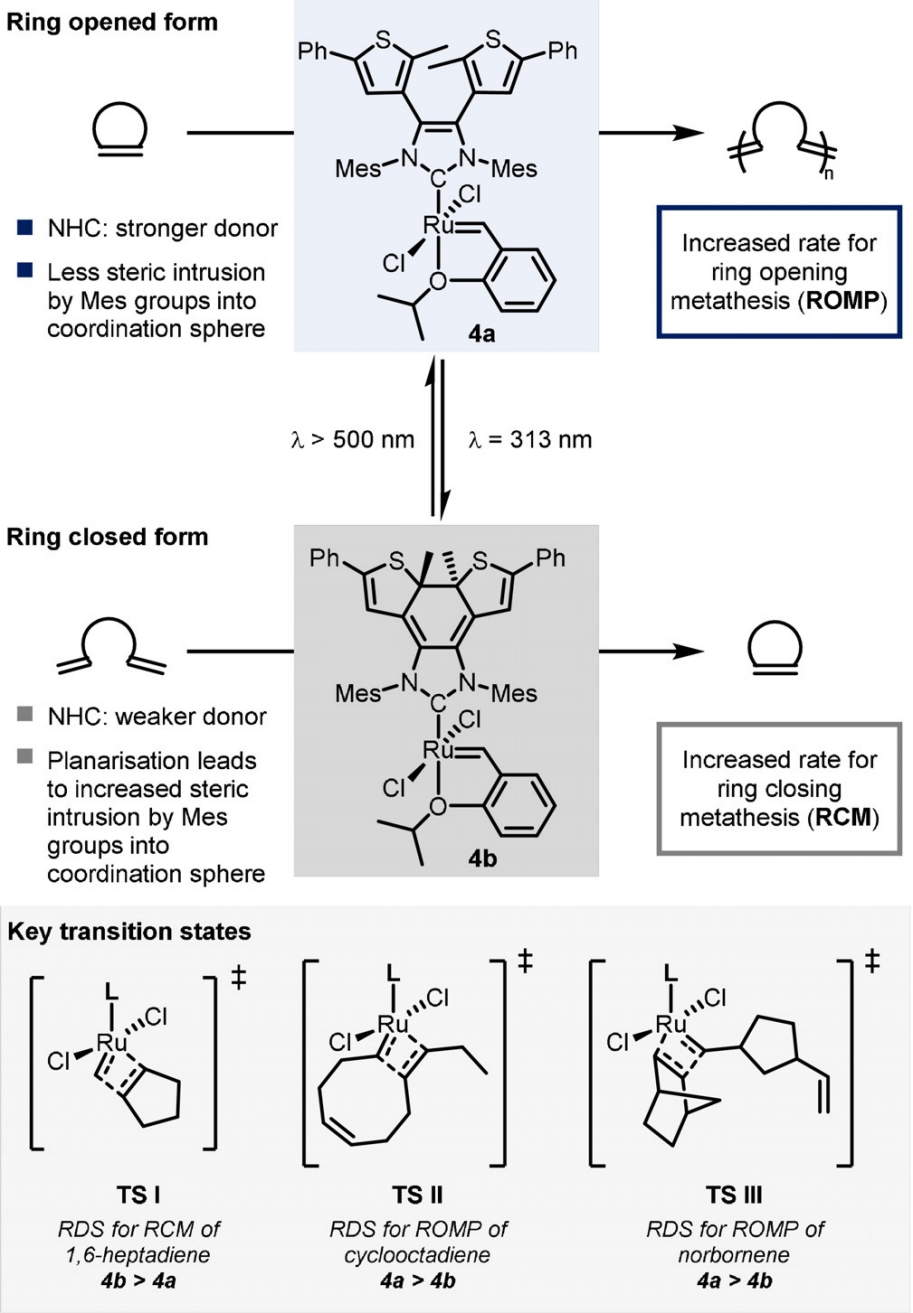
Light control over the function of a ruthenium metathesis catalyst.

The Bielawski group has investigated how the incorporation of a photochromic dithienylethene (DTE) moiety into an *N*‐heterocyclic carbene (NHC) ligand can modify its donicity.^[12]^ In this context, they developed the first photoswitchable olefin metathesis catalyst **4 a** based on the Hoveyda–Grubbs second‐generation catalyst (Scheme 4).^[13]^ This novel Ru^II^ catalyst has an NHC ligand which bears a photochromic DTE moiety. The UV/Vis spectrum exhibits an intense absorption band at 298 nm that was assigned to a combination of the n→π* transition of the *N*‐heterocycle and the π→π* transition of the aryl rings. Upon irradiation at 313 nm, a colour change occurs concurrent with decreased intensity of the band at 298 nm and the appearance of new absorption bands centred at 453 and 639 nm. Together with other experimental data (^1^H NMR shifts of the benzylidene resonance), this provided the evidence that electrocyclic ring closure had occurred to form an extended π‐conjugated system, **4 b**. Exposure to visible light (λ>500 nm) reverses this process, restoring the catalyst to the ring‐opened state **4 a**.

Having demonstrated the reversibility of the photo‐isomerisation process, the catalytic activity of the two forms of the catalyst were then investigated for both ring‐closing metathesis (RCM) and ring‐opening metathesis polymerisation (ROMP). It had been previously shown that the donating ability of this NHC ligand is significantly different in the two states. Calculations showed that this was important for RCM of 1,6‐heptadiene, where the rate‐determining step (RDS) was the transition state of the retro‐[2+2] cycloaddition (**TS I**, Scheme 4). In **4 a**, the NHC ligand is a stronger donor, which stabilises the Ru^IV^ metallacyclobutane preceding this transition state, resulting in a higher barrier for this step, and thus the process is faster for **4 b**.

For ROMP reactions, the ring‐opened form of the catalyst is more effective, but the reasoning is substrate dependent. For cyclooctadiene (COD), the increased steric bulk of the ring‐closed form of the catalyst (arising from increased intrusion of the mesityl groups as a result of planarisation) results in an unfavourable interaction in the rate‐determining retro‐[2+2] step (**TS II**, Scheme 4).

Conversely, for ROMP of norbornene, the calculated RDS is the [2+2]‐cycloaddition where the steric differences between the two forms of the catalyst are negligible. Here, the stronger donating ability of the ring‐opened form of the catalyst promotes the cycloaddition due to the stabilisation of the ruthenacyclobutane intermediate (via **TS III**, Scheme 4). Overall, this means the ring‐opened form of the catalyst **4 a** accelerates ROMP compared to the ring‐closed form **4 b**, whereas, in contrast, the ring‐closed analogue shows the opposite activity by increasing the rate of RCM. Up to 1.7‐fold rate enhancement is observed between the two photoswitchable catalytically active states of the complex.

Light‐responsive ligand motifs have also been applied for polymerisation reactions, where the external control over selectivity between different reactions or substrates can have a significant impact for modifying the composition of polymers.[^14^, ^15^] A number of other light‐responsive catalytic polymerisation systems have been reported[^16^, ^17^, ^18^, ^19^] and these systems are likely to play a significant role in developing materials of the future.[^20^, ^21^]

**Scheme 5:**
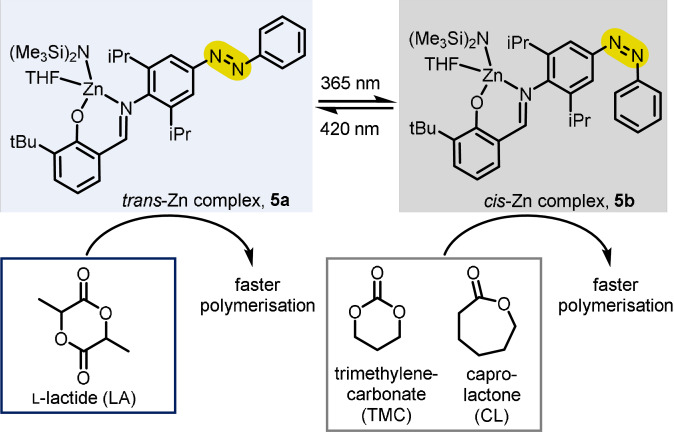
Azobenzene‐regulated polymerisation modes.

In 2019, Chen and co‐workers incorporated well‐explored azobenzene photoswitches into the ligand structure of salicyaldimine Zn^II^ complexes, which were investigated as polymerisation catalysts for ring‐opening polymerisation (ROP).^[22]^ Photoswitching was shown to be reversible in the case of the unsubstituted arene attached to the azobenzene (Scheme 5) in contrast to other substituted arenes. The authors investigated the ability of the two forms of the catalyst to discriminate between different lactone‐type monomers and thus alter the composition of the polymer based on an external stimulus. The *trans*‐Zn complex **5 a** can be isomerised to the *cis* form **5 b** with 365 nm light. The reverse process can be achieved with 420 nm light. Both isomers are active in ROP but show different reactivities, with the *trans*‐Zn complex being slightly more active for l‐lactide (LA) in contrast to most other monomers such as trimethylenecarbonate (TMC) and caprolactone (CL) which are more efficiently polymerised by the catalyst in its *cis* form.

Interestingly, isomerisation results in some discrimination during copolymerisation of a mixture of TMC and CL monomers. The initial rate of polymerisation for TMC for the *trans*‐Zn complex is 1.4 times faster than for CL; however, upon UV‐light‐induced isomerisation to the *cis*‐Zn complex, this changes to a 6.7‐fold rate difference, decreasing the amount of CL incorporation into the copolymer. It was speculated that a difference in the electronic effects between the two forms of the catalyst is responsible for the differences in activity that is observed, because the steric environment of the metal is largely unaffected by *cis*–*trans* isomerisation.

### 2.2 Ligand Photodissociation

Modifying the coordination sphere of a transition metal complex via ligand photodissociation has typically resulted in “on” and “off” states for catalytic transformations. However, it is also possible, though currently less predictable, to use this approach to give two different “on” states. By switching between open‐ and closed‐shell intermediates, completely different mechanisms can be favoured (e.g. a saturated coordination sphere might favour outer‐sphere electron transfer whereas free‐coordination sites would favour inner‐sphere substrate coordination).

**Scheme 6:**
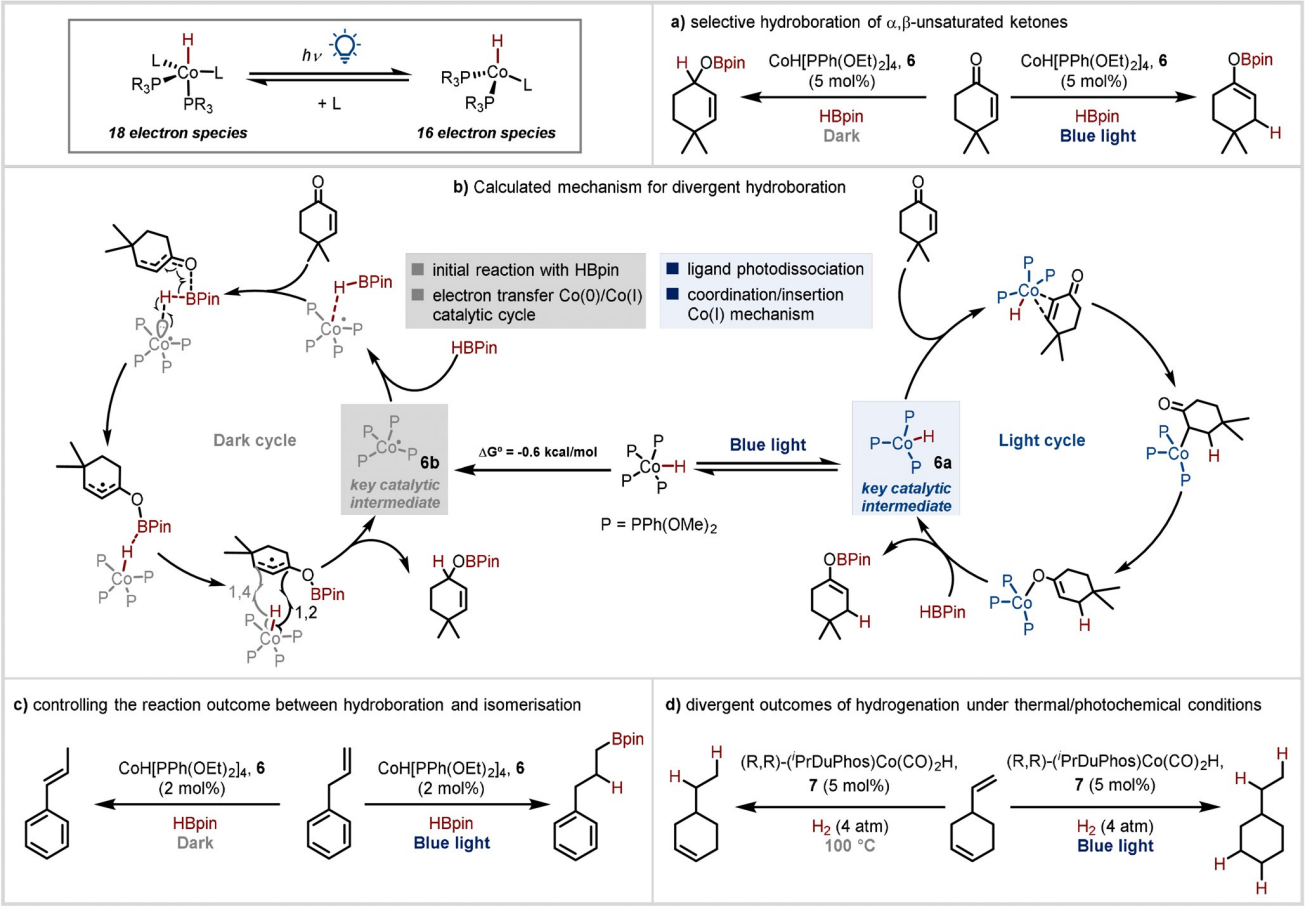
Photocontrolled cobalt catalysis via ligand photodissociation.

In this context, our group recently reported unique, photocontrolled chemoselective hydroboration reactions of α,β‐unsaturated ketones using the well‐defined transition metal complex CoH[PPh(OEt)_2_)]_4_ **6** in conjunction with pinacolborane.^[23]^ Notably, cyclic α,β‐unsaturated ketones were able to undergo selective 1,2‐ or 1,4‐hydroboration reactions depending on the absence or presence of visible light, respectively (Scheme 6 a). This approach also enabled the formation of cyclic boron enolates which had been a limitation of previous methodologies. These could be reacted directly with a range of aldehydes, in one‐pot aldol reactions, leading to the formation of *syn*‐aldol products. It was demonstrated that the divergent selectivity arises from distinctive mechanistic pathways in the dark and light. Irradiation with visible light triggers ligand dissociation (Scheme 6 b), thereby generating the coordinatively unsaturated 16‐electron cobalt(I) hydride complex **6 a** to which coordination of the substrate can occur. This results in a coordination/insertion type mechanistic pathway, with insertion into the C=C bond favoured, leading to the enolborates, and ultimately formation of the 1,4‐reduction product upon quenching. In the absence of light, however, the substrate is unable to coordinate at the saturated metal centre, resulting in a different starting point to the mechanism. Instead, an initial reaction with the pinacolborane and electron transfer to the starting material occurs to generate the Co^0^ intermediate **6 b**, which is an overall exergonic step. With the assistance of the starting material, the HBpin is activated by this Co^0^ intermediate, resulting in an HAT type mechanism.

Using a similar system, our group also reported the switchable reactivity of this system with terminal alkenes.^[24]^ Using the same cobalt(I) hydride complex, it was possible to switch between anti‐Markovnikov alkene hydroboration under visible light irradiation and alkene isomerisation in the dark via two distinct reaction pathways (Scheme 6 c).

Recently, a similar strategy has been reported by Chirik and co‐workers involving photodissociation of a CO rather than a phosphonite ligand.^[25]^ They focussed on the catalytic hydrogenation activity of a bench‐stable, coordinatively saturated cobalt(I) precatalyst **7**, (*R*,*R*)‐(iPrDuPhos)Co(CO)_2_H. Terminal, di‐ and trisubstituted alkenes, alkynes and carbonyls were investigated under both thermal and photochemical conditions. Hydrogenation occurs via divergent mechanistic pathways depending on the reaction conditions: upon heating to 100 °C, the reaction follows an HAT pathway facilitated by cleavage of the relatively weak Co−H bond (56 kcal mol^−1^). In contrast, irradiation of **7** with blue light leads to carbonyl ligand dissociation and formation of a 16‐electron complex, which undergoes a coordination–insertion sequence with the olefin involving closed‐shell intermediates. Generally, the photochemical method surpassed the thermal one in the case of hydrogenation of sterically hindered and electron‐rich substrates, with one example of a selectivity difference being described (Scheme 6 d).

These three examples underscore the potential of coordination‐sphere modification to impact the mechanistic pathway, resulting in divergent selectivity. Unusually, the radical mechanistic pathways occur under thermal/dark conditions rather than photochemical conditions, as might be expected. Nonetheless, improved reactivity was observed under photochemical conditions, demonstrating the ability of this approach to improve efficiency of catalysts.

**Scheme 7:**
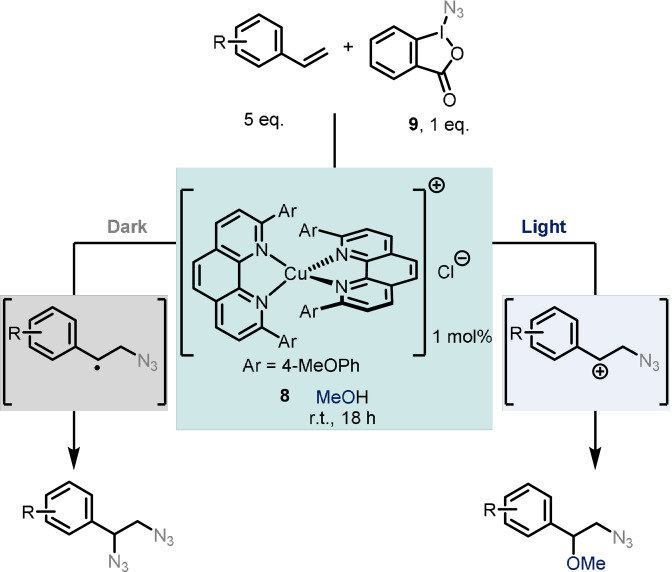
Light‐controlled switching between methoxyazidation and double azidation of styrenes.

Another example of photoswitchable behaviour was reported by Fumagalli et al., who investigated the behaviour of [Cu(dap)_2_]Cl (**8**) as the catalyst for an alkene difunctionalisation reaction.^[26]^ Here, the hypervalent iodine Zhdankin reagent **9** was used as a source of azide radical and a third polar component served as the nucleophile. The addition of azide to styrene‐type double bonds is followed by addition of a third component at the benzylic position. Light is able to control the degree of azidation in the following way: in the presence of visible light, oxidation of the benzylic radical occurs to yield the cation, which is subsequently quenched with the alcohol solvent resulting in methoxyazidation. In contrast, in the absence of light, double azidation occurs (Scheme 7). The authors suggest that this results from a change of mechanism to a radical chain process in this case, although more recent work notes that there is some evidence that ligand dissociation from Cu(dap)_2_Cl can occur upon light irradiation, which may lead to a switch in the mechanism between inner‐ and outer‐sphere.^[27]^ However, both alkene functionalisation reactions occur under mild reaction conditions at room temperature, and the reaction outcome is controlled solely by the presence or absence of visible light. It was shown that different substituent patterns are generally well‐tolerated, although more electron‐poor olefins do not yield the desired products under the reaction conditions.

### 2.3 Catalyst Excitation

**Scheme 8:**
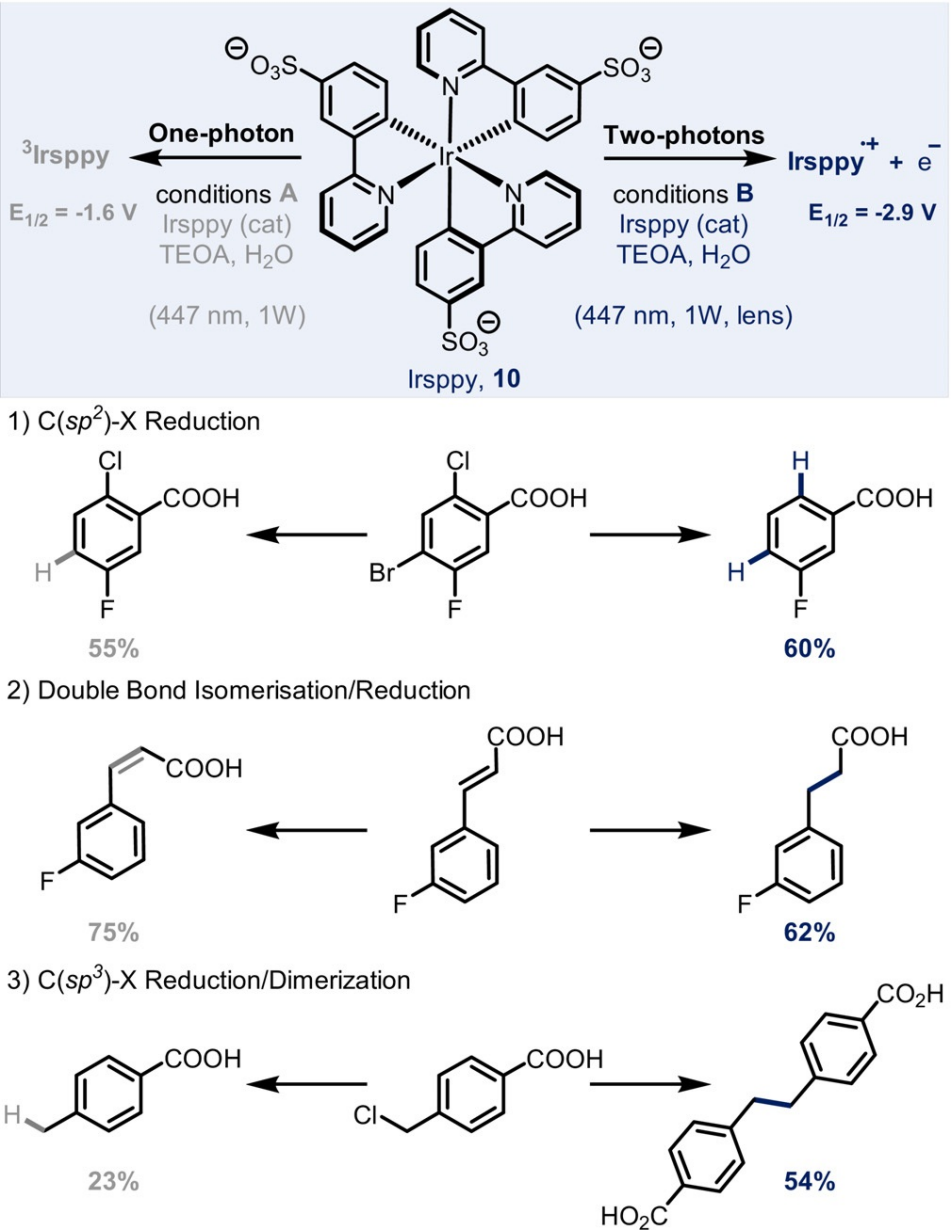
Controlling the reaction outcome by altering the intensity of light irradiation.

Modifying the irradiation wavelength can be a straightforward way of influencing the reactivity of a system.^[28]^ Alternatively, it is also possible to do so by altering the intensity of the irradiation.^[29]^ The effectiveness of this underexplored approach has been recently demonstrated by Kerzig and Wenger.^[30]^ They reported that the switching from the one‐ to two‐photon activation of an Ir‐based photocatalyst can lead to the formation of different products under very similar conditions. The modulation of the light intensity per area is realised with inexpensive optics commonly used for beam collimation. The water‐soluble photocatalyst Irsppy (**10**), previously designed by the same group, can absorb a photon under blue light irradiation to form the corresponding excited triplet state, which can be engaged in dehalogenation and isomerisation reactions (Scheme 8, left). If another photon is absorbed within the lifetime of the excited state (and this can be achieved by using a light source with higher intensity per irradiation area), hydrated electrons (e_aq_^.−^) are generated. In this case, the system exhibits different behaviour that leads to the formation of different products compared to those formed by the one‐photon pathway (Scheme 8, right).

The authors focused their attention on three processes: 1) the selective reduction of C(sp^2^)–halogen bonds in an aromatic system; 2) the *trans‐*‐*cis* isomerisation of olefins, rather than their hydrogenation; and 3) the reductive dehalogenation of a benzyl chloride, in contrast to its dimerisation. For each process, the different reactivity is a result of the different properties of ^3^Irsppy and e_aq_^.−^. For process 1), the selectivity is controlled by the redox potentials of the two species: while ^3^Irsppy (*E*_1/2_=−1.6 V) is only able to reduce C−Br bonds, e_aq_^.−^ (*E*_1/2_=−2.9 V) is able to activate the more challenging C−Cl bond. In the case of process 2), the alkene isomerisation is induced by a triplet–triplet energy transfer (TTET) whereas the reduction occurs via a direct electron transfer (ET). Lastly, in system 3), the starting material is consumed much faster in the two‐photon conditions; this means that the local concentration of benzyl radical is high enough to favour the dimerisation over the simple dehalogenation.

### 2.4 Aggregation State

**Scheme 9:**
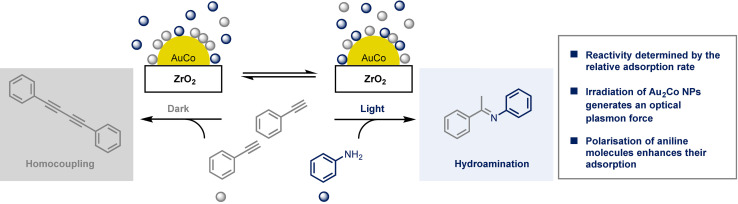
A photoselective reaction as a result of contrasting adsorption properties.

The outcome of a reaction can also be altered by influencing the rate of adsorption and desorption onto a heterogeneous catalyst. Sarina and co‐workers reported that the alkyne hydroamination reaction catalysed by Au_2_Co alloy nanoparticles leads to the expected cross‐coupling product, an imine, when carried out under visible light irradiation but in the dark mainly homocoupling of the alkyne is observed (Scheme 9).^[31]^

The switch in the selectivity arises from the different rate of adsorption of the two substrates on the nanoparticle surface and it can be altered under low‐flux visible light irradiation. The light affects the system in two ways that contribute to the formation of the cross‐coupling product: 1) light enhances the adsorption of aniline to the catalyst surface; 2) light also weakens the adsorption of the alkyne. It had been previously reported that gold nanoparticles can display catalytic activity due to the surface plasmon resonance effect, whereby the oscillation of conduction electrons resonates with the electromagnetic field of the incoming light, thus enhancing local magnetic fields on the surface of the nanoparticle, and ultimately resulting in excited electrons.^[32]^ For this example, a plasmon‐generated optical plasmon force might explain why aniline adsorption is favoured in the light, but this force is expected to have a negligible effect on a system irradiated with a low‐flux visible light source such as the one used in this study. However, the interaction between the alloy and the substrate is also influenced by the polarizability of the substrate. Under these conditions, aniline can be converted to its electronically excited state, which presents a considerably higher polarizability. The optical plasmon force experienced by the excited aniline is therefore stronger and it is postulated to be sufficient to overcome the Brownian motion of the excited molecules, leading to the adsorption onto the alloy surface. On the other hand, phenylacetylene does not absorb visible light and therefore it is not affected by the same force.

**Scheme 10:**
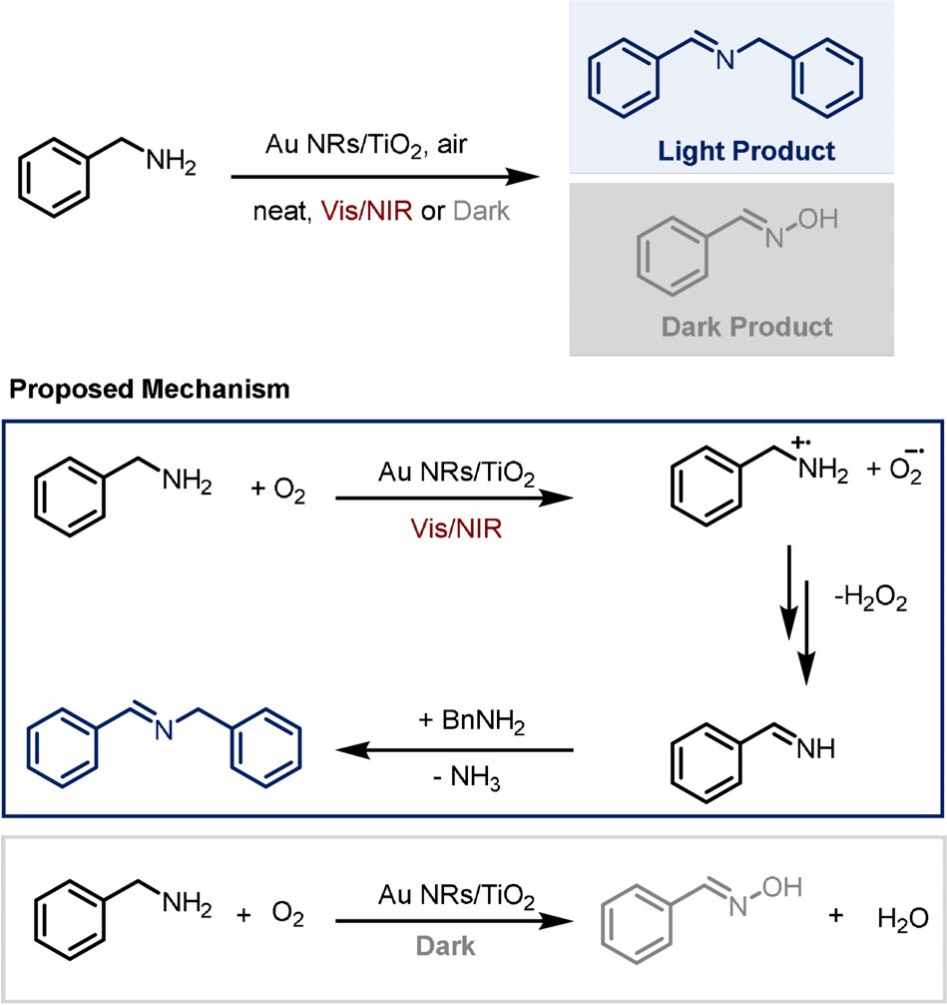
Divergent photochemical oxidation of benzylamines.

Zhao and co‐workers studied the catalytic activity of gold nanorods (Au NRs) supported on TiO_2_ nanofibers.^[33]^ After the synthesis and characterisation of this novel material, the authors turned their attention to a potential synthetic application. They investigated the photochemical homocoupling of benzylamines under air and in solvent‐free conditions. The reaction leads to the formation of a benzylidenebenzylamine as the major product and benzaldoxime, as a minor byproduct when carried out under visible light or near‐IR irradiation. Interestingly, the selectivity of the reaction is partially reversed when it is carried out in the dark (Scheme 10). When benzaldoxime is used as reactant in the “light conditions” no homocoupling is observed, which indicates that this species is not an intermediate in the imine formation process. It is therefore likely that two different reaction pathways are responsible for the different selectivity. EPR studies suggest that O_2_^.−^ is present in the light pathway but not in the dark and, unsurprisingly, no reactivity is observed in absence of oxygen. According to the proposed mechanism, when oxygen and the starting material are adsorbed onto the catalyst surface, the light triggers the transfer of hot plasmonic electrons that leads to the formation of O_2_^.−^ and an *N*‐centred radical cation. This cation can be further oxidised to an aldimine that condenses with a molecule of benzylamine, generating the imine and ammonia. In the dark, benzylamine is directly oxidised to generate the aldoxime.

## 3 Substrate Modification

Another approach to alter the selectivity of transition metal catalysed reactions is to use light to modify the substrate. This could involve altering the geometry of the starting material, or activating a certain functionality from the ground state such that it becomes more reactive than another functional group which reacts in preference in the dark.

**Scheme 11:**
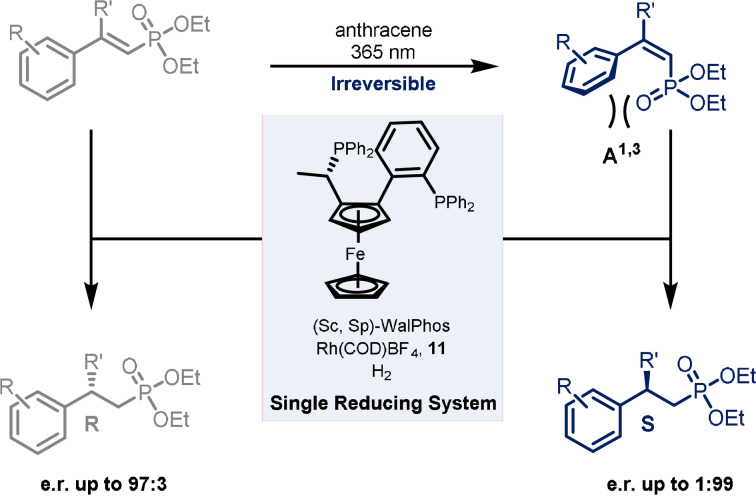
Enantiodivergent reductions using a single enantiopure reduction catalyst via alkene isomerisation.

For instance, light has been used as a trigger to switch the enantioselectivity of a reaction by acting only on the substrate, where commonly a change in the configuration of the catalyst would have been required. Gilmour and co‐workers reported the enantiodivergent synthesis of β‐chiral phosphonates starting from stereodefined α,β‐unsaturated phosphonates using a single enantiopure catalyst (Scheme 11).^[34]^ The reduction, carried out under 10 atm of H_2_ and catalysed by the rhodium complex **11**, can be preceded by the photoisomerisation (at 365 nm) of the double bond. This is achieved by selective energy transfer using inexpensive anthracene as the photocatalyst. *E*‐vinyl phosphonates are irreversibly isomerised to the *Z* isomer due to the 1,3‐allylic strain that inhibits the re‐excitation by weakening the conjugation between the π system of the arene ring and the double bond. The nature of the substituents R and, in particular, R′ are important to achieve a high *Z*/*E* ratio; indeed, when R=R′=H, the photostationary state consists of a 1:1 mixture of the two geometric isomers. Moreover, the efficiency of the isomerisation is lost when R is an electron‐rich group (such as a methoxy). This can be explained considering a greater contribution of resonance forms that can facilitate the rotation of the C−C bond, leading to the *E* isomer after relaxation. The subsequent rhodium‐catalysed reduction occurs with high stereospecificity, affording the two enantiomeric products with e.r. up to 99:1. When the *E*‐phosphonate is reduced, the (*R*)‐enantiomer is formed, whereas if the starting material is first converted to the *Z* isomer, the (*S*)‐enantiomer is obtained. Compared to more traditional approaches to enantiodivergent catalysis, this method has the advantage of using a single enantiomer of the catalyst. Normally the switch in enantioselectivity is achieved by using the enantiomer of the catalyst, but sometimes accessing it might not be straightforward.

**Scheme 12:**
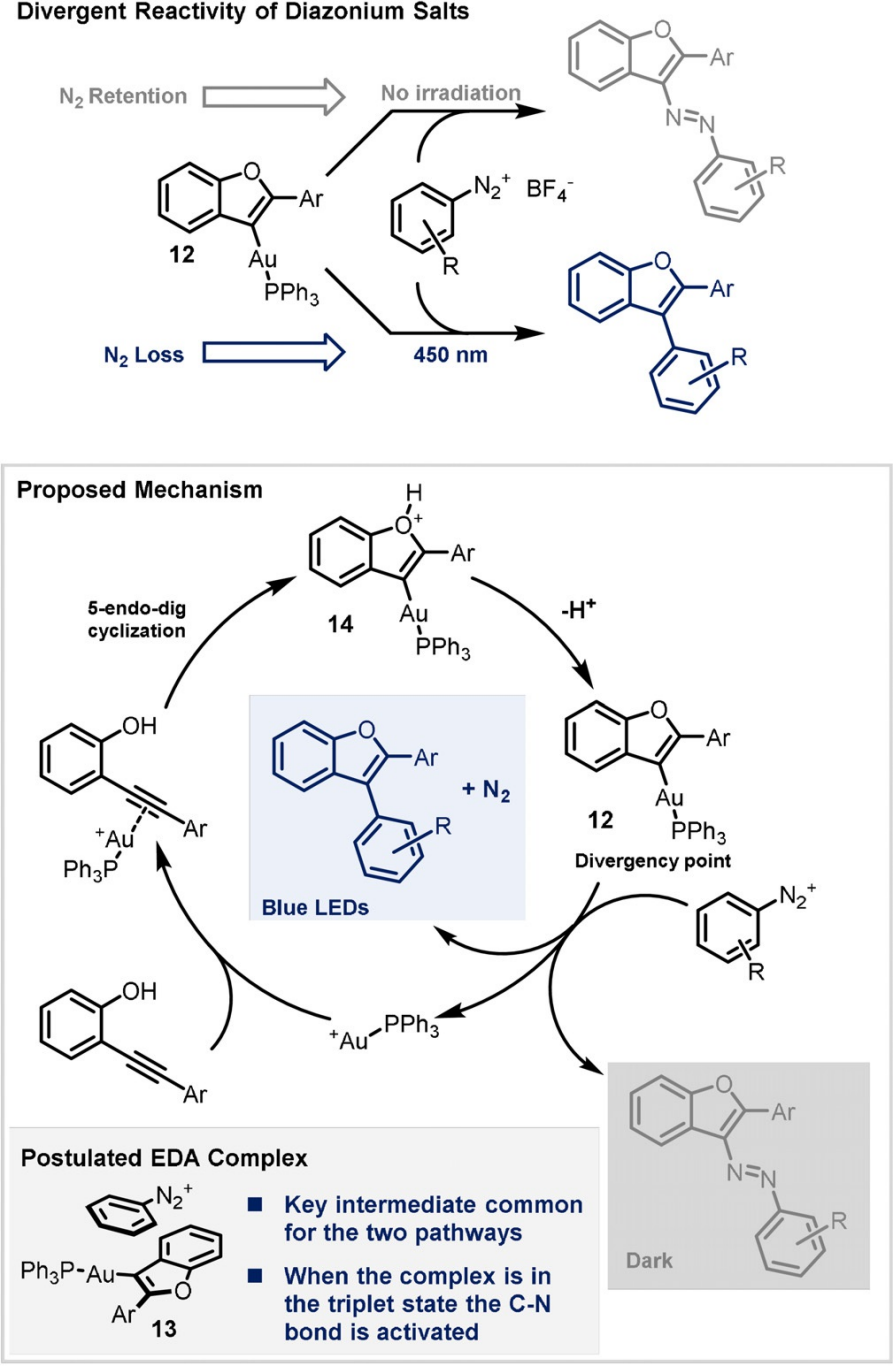
Chemodivergent reactivity using gold catalysis.

In a different context, Hashmi, Klein and co‐workers demonstrated that aryldiazonium salts can show chemodivergent reactivity under the action of gold catalysis, depending on whether the reaction is carried out under blue LED irradiation (450 nm) or in the absence of a light source (Scheme 12).^[35]^ In the first case, the reaction of the diazonium salt with *o*‐alkynylphenols leads to arylated benzofurans; in the latter, to substituted azobenzofurans. The authors carried out extensive (experimental and computational) mechanistic studies to gain insight into the origin of the divergency. Based on their results, the formation of a key vinyl Au^I^ complex **12** was postulated. This species can form EDA complex **13** with the diazonium salt, and irradiation of this complex leads to N_2_ extrusion, resulting in the formation of a new C−C bond. In absence of a light source, the diazo moiety is retained and the diazonium salt behaves as an *N*‐electrophile. Apart from the last step, the mechanisms proposed for the two transformations are equivalent. The initial π‐coordination of the Au complex with the alkyne moiety activates the triple bond towards a 5‐*endo*‐dig cyclisation, in which the oxygen functions as nucleophile. The resulting intermediate (**14**) is deprotonated, generating vinyl Au^I^ complex (**12**) that can then evolve into one of the two products, depending on the conditions.

In one final example, Lemcoff and co‐workers investigated a chromatic orthogonal catalytic process in which two photochemical processes, triggered by light of different energy, are combined: photocleavage of a supersilyl protecting group at 254 nm and the activation of the dormant Ru catalyst **15 a** for RCM reaction at 350 nm.^[36]^ Interestingly, the supersilyl group can influence the selectivity of ring‐closing metathesis reactions, altering the preferred ring size that is formed compared to that with the unprotected alcohol starting material. This opened up the possibility of developing a noncommutative sequence, whereby the selectivity of the reaction would be dictated by the order of the sequence of light irradiation of different wavelengths.

**Scheme 13:**
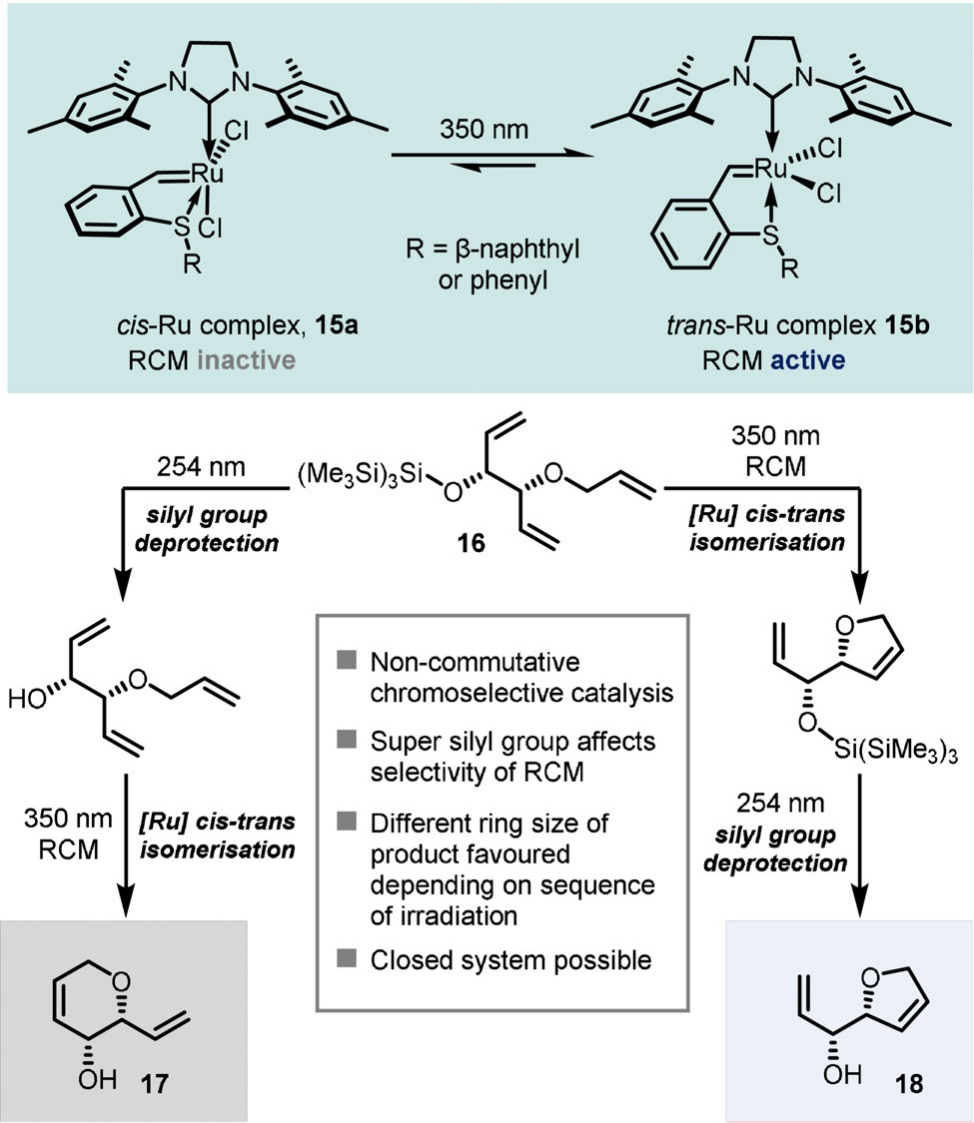
A non‐commutative reaction sequence controlling product selectivity depending upon the order of wavelengths used.

In the first sequence (Scheme 13, left), the supersilyl‐protected triene **16** was irradiated first at 254 nm, resulting in cleavage of the supersilyl group, with the metathesis catalyst remaining intact but inactive. Notably, the selection of solvent (1,1,2,2‐tetracholorethane) was important to allow deprotection of the supersilyl group in the presence of the ruthenium catalyst, and a less‐active precatalyst (where R=phenyl) was more effective in the one‐pot sequence due to lessened decomposition. Subsequent irradiation of the reaction mixture (together with addition of CD_2_Cl_2_ to enhance the *cis*–*trans* isomerisation of the Ru catalyst) led to a RCM reaction which provided the six‐membered dihydropyran structure **17** in 6:1 preference to five‐membered dihydrofuran **18**. In contrast, for the second sequence (Scheme 13, right), the reaction mixture was first irradiated with 350 nm light, which led to preferential formation of a five‐membered ring via RCM due to the bulkiness of the protected alcohol. Irradiation at 254 nm led to the cleavage of the silyl protection group, giving product **18** as the major product (30:1 ratio with **17**).

An important prerequisite for developing this orthogonal process was that the light‐sensitive supersilyl protecting group remains intact upon irradiation with 350 nm light, and the RCM catalyst remains largely untouched under irradiation with 254 nm light. The development of non‐commutative sequences such as this holds exciting potential for the development of divergent synthetic routes controlled solely by light.

## 4 Summary and Outlook

To conclude, it has been shown that light can influence the course of transition metal catalysed reactions in a range of different ways. Excitation of the catalyst, either directly or of the ligand, can result in alteration of the steric or electronic properties of the active catalyst. Alternatively, light‐induced modification of the substrate, for example via isomerisation or deprotection, can also result in divergent selectivity or reactivity. These approaches open up the possibility to access multiple different functions for a single catalytic system. Together with the advances in automation technology in synthetic chemistry, this has potential to lead to more efficient, controlled sequences of catalytic transformations. Truly reversible, switchable processes could be combined in production lines to allow efficient and swift diversification of common intermediates.

Despite the current limited range of examples on this topic, the reported approaches encompass a broad array of different strategies. Due to its non‐invasive character and ease of set‐up, light remains an easy and appealing way to modify the reactivity of transition metal catalysts. Without doubt, there are many opportunities going forward to design light‐responsive catalysts which will allow us precise control over selectivity of reactions in a way that was not previously possible. New work in this area is likely to arise both from exquisitely designed strategies, relying on careful ligand design, but also from serendipitous discoveries. Future research will undoubtedly lead to the discovery of many new divergent modes of catalysis which are poised to have applications across synthetic chemistry.

## Conflict of interest

The authors declare no conflict of interest.

## Biographical Information

*Danijela Lunic was born in Novi Sad (Serbia) and studied for her BSc and MSc in chemistry at the University of Novi Sad before obtaining a further MSc degree from the Erasmus Mundus program SERP+. In October 2020, she moved to RWTH Aachen University to begin PhD research under the supervision of Dr. Teskey. Her work is focused on the development of novel hydride‐catalysed methodologies*.

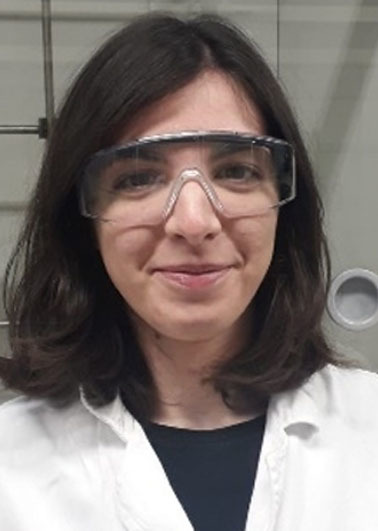

## Biographical Information

*Enrico Bergamaschi was born in Genoa (Italy) and received his undergraduate education at the University of Genoa, with his master thesis focused on the development of new photochemical methodologies. Since April 2019, he has been a PhD student at RWTH Aachen University under the supervision of Dr. Teskey. His research projects are centred around the combination of visible light and transition metal catalysis*.

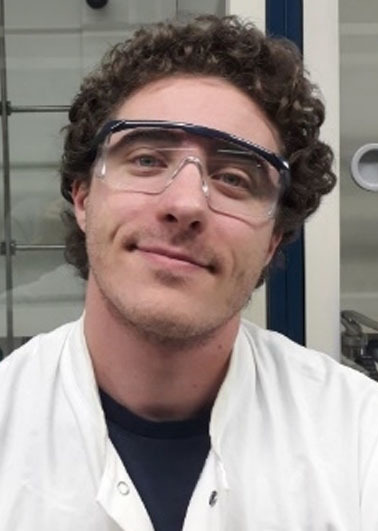

## Biographical Information

*Christopher J. Teskey studied Natural Sciences at the University of Cambridge (UK) before moving to the University of Manchester for a PhD in Organic Chemistry under the supervision of Prof. Michael Greaney. After a postdoctoral spell with Prof. Nuno Maulide at the University of Vienna, he began his independent career as a Junior Research Group Leader at RWTH Aachen University in 2019 funded by a Liebig Fellowship. His research interests revolve around hydride catalysis and photochemistry*.